# A dataset on Whatsapp groups effectiveness in inducting first years to university

**DOI:** 10.1016/j.dib.2024.110456

**Published:** 2024-04-21

**Authors:** Tshepo Rabotapi, Samson Matope

**Affiliations:** Walter Sisulu University, Nelson Mandela drive, Mthatha, South Africa

**Keywords:** Student orientation, Dropout, Academic support

## Abstract

WhatsApp as the most popular instant messaging technology that has created opportunities for online cooperation and teamwork among students in the university context [1] and allows students to communicate even outside school hours [2]. Transitioning from high school to university can be a challenging experience for many students, particularly those who are entering a new academic atmosphere with greater autonomy and higher workload [3]. This data publication [4] presents a dataset capturing the perceptions of first-year university students on the utilization of WhatsApp as a means of continuous induction into university life. The dataset includes responses from participants on 7 background questions and 14 Likert scale questions, measuring agreeableness on the academic dimension of the academic dropout wheel [5].

The questionnaire aimed to investigate the effectiveness of WhatsApp groups established by academic support staff in facilitating the integration of first-year students into both formal academic structures and informal social systems within the campus. The data may offer valuable insights into the potential benefits of WhatsApp as an educational tool and its impact on fostering a positive academic environment, ultimately helping to reduce dropout rates in institutions of higher learning.

Researchers, educators, and policymakers can utilize this dataset to gain a deeper understanding of the role that WhatsApp can play in enhancing student engagement and promoting successful integration into university life. By harnessing the information from this dataset, institutions can develop targeted strategies to provide effective support systems for first-year students, thereby promoting academic success and retention.

Specifications Table

**Table:** 

Subject	Social Sciences
Specific subject area	Educational Technology and Student Engagement in Higher Education. It discusses the use of WhatsApp as an instant messaging technology to enhance communication, collaboration, among first entering university students. The primary focus is on how WhatsApp facilitates the transition and integration of first-year students from high school to university by providing a means of continuous induction into university life.
Data format	Raw, Analysed, Filtered
Type of data	Table
Data collection	The data was acquired through google forms questionnaire, the form was created As first year entrants registered to the university online from their homes, they were sent links to join WhatsApp groups administered by campus educational technologist. The administrator then allowed students to interact with each other to find directions to various off campus residences, best times, and best ways to travel, and demystify perceptions of higher education spaces. Time and time the administrator would step in to keep the group in check, teach about digital literacies, and give announcements about the university. This allowed other psychosocial support departments to also make announcements and let themselves known to first year students. A year later a questionnaire crafted based on academic elements the academic dropout wheel [5] and sent to the group via the WhatsApp groups. after 20% of the total cohort answered, the form was closed for analyses. the questionnaire is provided as a supplementary file
Data source location	The data was collected at Komani Campus of Walter Sisulu University in South Africa. Data is stored at Mendeley Data.
Data accessibility	Repository name: Mendeley Data Data identification number: 10.17632/tycj43cfnk.2 Direct URL to data: https://data.mendeley.com/datasets/tycj43cfnk/2
Related research article	

## 1 Value of the Data

The data contains valuable insights about student experiences and interactions through WhatsApp groups. This information can be beneficial in several ways:

• Enhancing Student Engagement: Researchers can analyze the relationship between belongingness [6,7] (feeling welcomed and connected) and increased participation in extra-curricular activities [8] through WhatsApp groups. This insight can guide universities in fostering a supportive environment which can help with student retention.
• Improving Academic Performance: The data on how WhatsApp groups aid in finding academic support, valuable class information, and assisting in class performance can help researchers further understand the impact of digital platforms on students' learning outcomes [9].
• Social Integration: this can add to literature about the role of WhatsApp groups in forming positive relationships, trust [10], and a safe space and can help universities create strategies to enhance students' social integration and overall well-being.
• Effective Communication Channels: The data highlights how WhatsApp groups help students navigate university life, find events, and connect with academic resources [11]. Researchers can advise institutions on optimizing communication platforms for better student engagement.
• Adaptation to University: The insights add on students adjusting to university studies, finding academic support [11], and expressing oneself freely on WhatsApp groups can aid researchers in identifying factors that contribute to successful student transitions.

## 2 Data Description

The dataset is structured as follows:

Files: PDF File (data coding.pdf): This file provides an explanation of the coding process used for the dataset. It details how the coded data sheet in the Excel file was generated, offering insight into the transformation of raw data into meaningful categories.

Excel File (Whatsap group effectiveness Measure (Responses).xlsx): This Excel file contains two sheets: one for raw uncoded data and the other for coded data.

Sheets within Excel File: Sheet (Raw Data): This sheet contains the raw, unprocessed data collected through the Google Forms questionnaire distributed within the WhatsApp groups. Each row represents a student's response, with columns capturing various aspects of their experiences, perceptions, and interactions within the university environment.

Sheet 2 (Coded Data): The coded data sheet presents the transformed and categorized data derived from the raw responses. This categorization aims to highlight patterns, trends, and key insights relevant to student engagement, belonging, and interaction. The PDF file (data coding.pdf) provides the coding guide for this transformation process.

These files collectively form a dataset that offers valuable information about student engagement, interaction, and experiences within university settings, particularly through WhatsApp groups. The dataset's focus on digital communication platforms provides insights into how technology impacts academic engagement, social integration, and overall student well-being.

## 3 Experimental Design, Materials and Methods

The data acquisition process involved several steps and tools to ensure comprehensive and ethical data collection:

Questionnaire Design: we developed a questionnaire based on a 7-point agreeableness Linkert scale, adapted from Naaman's academic dropout wheel [5]. Likert scales according to Robinson [12] are popular scale formats to measure public opinion on any issue, with the 7-point scale used to test how strongly the participants agree or disagree with an opinion statement from 1 (=strongly agree) and 7(strongly disagree) and 3 being neutral feeing.

The questionnaire which is attached as a supplementary file, focused on the academic dimension of the academic dropout wheel [5] which has 4 key areas: Feeling of belonging, extra-curricular participation, academic performance and satisfaction and academic integration. The academic dimension was selected due to nature of researchers work, which is academic support. The 4 key areas were also identified as some of the benefits of orientation by Evenson [13] and Altschwager et al. [14] Additionally, the first section gathered biographical information of the participants.

Platform and Ethical Clearance: The questionnaire was created on Google Forms, accompanied by a statement of ethical clearance at the beginning of the survey. This ensured that participants were informed about the purpose of the study and their rights, adhering to university ethical guidelines and obtaining participants' consent [15].

Participant Recruitment and Availability: The survey was open for two weeks, targeting first-year students of the 2022 academic year. The survey link was posted on various first year WhatsApp groups, the QR code linking to the form was printed and posted in notice boards across the campus and students were also encouraged by visiting lecturer halls during class and explaining the aim of the research.

Data Collection: We distributed the survey to first-year university students through WhatsApp groups managed by campus educational technologists, these groups provided a platform for students to engage and share information. We also printed and posted QR codes linking to the survey on various notice boards on campus. Participants accessed the survey link, completed the questionnaire, and submitted their responses via the Google Forms platform.

Data Preparation and Storage: The collected data were stored securely within the Google Forms platform to maintain participant confidentiality.

Data Analysis: Raw data were transferred from Google Forms to an Excel file consisting of two sheets: one for raw uncoded data and another for coded data. The coding process involved transforming the raw data into categorized insights based on the Likert scale responses. The coding process was detailed in the provided PDF document (data_coding.pdf).

The results show an increased Feeling of belonging as with study by rambe [16] and Bouhnik [2] extra-curricular participation, better academic performance, satisfaction and academic integration which reiterates study by Cetinkaya [17] and Rambe [16].

Higher education institutions can benefit on the use of social media tools like WhatsApp to extend orientation of first years to a longer period. The results show that the groups increased a feeling of belonging to the university by students, knowledge and trust of the various academic support units. The information shared on social media tools assist in joining the students into cocurricular activities, which help in integration them more into university. When freely expressing themselves, students can adjust to university and perform better in various classes. This includes being able to get class times, clarification of content.

Dataset and Results: Coded data, as well as the detailed explanation of the coding process, were made available in the dataset files (Excel and PDF) for further analysis and interpretation.

The comprehensive process involved the design of a purposeful questionnaire, ethical considerations, participant recruitment through specific communication channels (WhatsApp groups), and secure data storage. This approach ensured that the data collected accurately represented the experiences, perceptions, and engagement of first-year university students within the context of the academic environment and the impact of WhatsApp to their induction.

## Limitations

Sample bias: this study targeted first-year university students of 2022 academic year, which may not represent a broader student population. This could limit generalisability of findings.

**Fig. 1:**
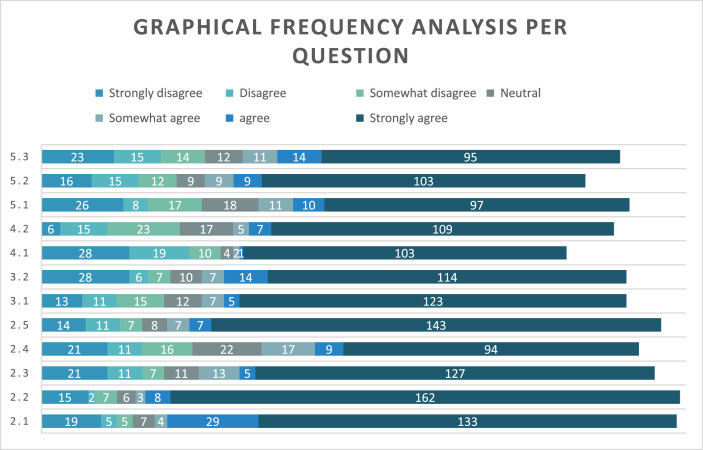
Graphical frequency analysis per question.

Relying on self-reported data: Participants responses may not reflect their actual experiences or behaviours, leading to inaccuracies in findings Fig. 1, Table 1.

**Table 1:** Frequency analysis per question

Question number	2.1	2.2	2.3	2.4	2.5	3.1	3.2	4.1	4.2	5.1	5.2	5.3
Strongly disagree	19	15	21	21	14	13	28	28	6	26	16	23
Disagree	5	2	11	11	11	11	6	19	15	8	15	15
Somewhat disagree	5	7	7	16	7	15	7	10	23	17	12	14
Neutral	7	6	11	22	8	12	10	4	17	18	9	12
Somewhat agree	4	3	13	17	7	7	7	2	5	11	9	11
agree	29	8	5	9	7	5	14	1	7	10	9	14
Strongly agree	133	162	127	94	143	123	114	103	109	97	103	95

## Ethics Statement

This work was carried out in accordance with Protection of Personal Information Act of 2021 and informed consent was obtained from all participants. The study was approved by AUTHORITY OF UNIVERSITY RESEARCH ETHICS COMMITTEE certificate number FEDREC15-06-23-3 from Walter Sisulu University.

## CRediT authorship contribution statement

**Tshepo Rabotapi:** Conceptualization, Methodology, Investigation, Writing – original draft, Visualization. **Samson Matope:** Validation, Formal analysis, Resources, Writing – review & editing.

## Data Availability

Whataspp Effectiveness measure (Original data) (Mendeley Data).

## Appendix Supplementary materials

Image, application 1

## References

[1] Maphosa V., Dube B., Jita T. (2020). A UTAUT evaluation of whatsapp as a tool for lecture delivery during the COVID-19 lockdown at a Zimbabwean University. Int. J. Higher Educ..

[2] Bouhnik D., Deshen M. (2014). WhatsApp goes to school: mobile instant messaging between teachers and students. J. Info. Technol. Educ..

[3] McMillan W. (2013). Transition to university: the role played by emotion. Eur. J. Dent. Educ..

[4] Rabotapi T., Matope S. (2023). WhatsApp Effectiveness measure. Mendeley Data.

[5] Naaman H. (2021). The academic dropout wheel analyzing the antecedents of higher education dropout in education studies. Eur. Educ. Res..

[6] M.A. Fabris, M. Settanni, C. Longobardi, D. Marengo, Sense of Belonging at School and on Social Media in Adolescence: Associations with Educational Achievement and Psychosocial Maladjustment, Child Psychiatry Hum Dev (2023). 10.1007/s10578-023-01516-x. PMC11485285 36920688

[7] Allen K.-A., Gray D.L., Baumeister R.F., Leary M.R. (2022). The need to belong: a deep dive into the origins, implications, and future of a foundational construct. Educ. Psychol. Rev..

[8] Calanoga M.C., Tattao L., Julian C., Malana M. (2020). English performance of students’ and their participation to extra-curricular activities: bases for intervention programs. Asian EFL J..

[9] A. Singh, S. Sharma, M. Paliwal, Adoption intention and effectiveness of digital collaboration platforms for online learning: the Indian students’ perspective, Interactive Technology and Smart Education 18 (2021) 493–514. 10.1108/ITSE-05-2020-0070.

[10] Kuru Ozan, Campbell Scott W., Bayer Joseph B., Baruh L., Ling R. (2022).

[11] Ochuole O.Lydia, Bepeh U.S., Okpe O.M., Lwhu A.A. (2023). Perceived influence of whatsapp usage on students’ academic achievement in the university of cross river state. Int. J. Multidisciplinary Res. Anal..

[12] J. Robinson, Likert Scale, in: Encyclopedia of Quality of Life and Well-Being Research, Springer Netherlands, Dordrecht, 2014: pp. 3620–3621. 10.1007/978-94-007-0753-5_1654.

[13] K. Evensen, Benefits and level of satisfaction a first-year orientation benefits and level of satisfaction a first-year orientation program delivers for freshmen in college program delivers for freshmen in college, 2017. https://pillars.taylor.edu/mahe/92.

[14] Altschwager T., Dolan R., Conduit J. (2018). Social brand engagement: how orientation events engage students with the university. Australasian Market. J..

[15] Arellano L., Alcubilla P., Leguízamo L. (2023). Ethical considerations in informed consent, in: ethics - scientific research, ethical issues, artificial intelligence and education [Working Title]. IntechOpen.

[16] Rambe P., Chipunza C. (2013).

[17] Cetinkaya L. (2017). The impact of whatsapp use on success in education process. Int. Rev. Res. Open Distance Learn..

