# Supplementary material for: A dataset on Whatsapp groups effectiveness in inducting first years to university
Source: Data Brief. 2024 Apr 21;54:110456. doi: 10.1016/j.dib.2024.110456 (PMC11070691; doi:10.1016/j.dib.2024.110456)
Supplement: Supplementary file 1 [file mmc1.pdf]

Questionaire

**First question: biographical background**						
1. My Gender is	Male, Female, Non-Binary, prefer not to say					
2. I stay in	Residence, Home, Rented accommodation					
3. I am funded by	NSFAS, Self-Funded, Bursary/other					
4. Are you on Whatsapp Groups created by DLT?	Yes/No					
5. Does your home/Res have a good internet strength?	Yes/No					
6. Do you have a laptop or smartphone (choose all you have)	Laptop, Smartphone, Both					
7. Did you have family, friends, or people you knew at this campus before coming to campus?	Yes/No					
**Second question: feeling of belonging**	Strongly Disagree					Strongly agree
1. was I felt welcomed at the university since I joined the WhatsApp groups	7	6	5	4	3	2	1
2. WhatsApp groups help you to feel connected to academic support (eLearning, Pals, academic advising)	7	6	5	4	3	2	1
3. From WhatsApp groups, I feel that people at DLT (Directorate of Learning and Teaching) understand me as a person	7	6	5	4	3	2	1
4. I have made positive relationships with my peers from the WhatsApp groups.	7	6	5	4	3	2	1
5. The DLT WhatsApp groups are a safe, trustable space.	7	6	5	4	3	2	1
**Third question: extra-curricular participation**							
1. WhatsApp groups helped me find school events and activities easily.	7	6	5	4	3	2	1
2. Sub question 2: I have joined extra-curricular activities from WhatsApp group postings.	7	6	5	4	3	2	1
**Fourth question: academic performance and satisfaction**							
1. WhatsApp groups assistance helped me to perform better in class.	7	6	5	4	3	2	1
2. WhatsApp groups have helped me find my way around university.	7	6	5	4	3	2	1
3. I got valuable information about class from WhatsApp groups.	7	6	5	4	3	2	1
